# Biomechanical integrity score of the female pelvic floor

**DOI:** 10.1007/s00192-022-05120-w

**Published:** 2022-03-01

**Authors:** Vladimir Egorov, Heather van Raalte, Peter Takacs, S. Abbas Shobeiri, Vincent Lucente, Lennox Hoyte

**Affiliations:** 1 Advanced Tactile Imaging, 1457 Lower Ferry Rd, Trenton, NJ 08618 USA; 2 Princeton Urogynecology, Princeton, NJ USA; 3 Eastern Virginia Medical School, Norfolk, VA USA; 4 INOVA Fairfax Hospital, Falls Church, VA USA; 5 The Institute for Female Pelvic Medicine & Reconstructive Surgery, Allentown, PA USA; 6 The Pelvic Floor Institute, Tampa, FL USA

**Keywords:** Pelvic organ prolapse, Tissue elasticity, Pelvic support, Pelvic muscle strength, Muscle relaxation, Muscle mobility, Biomechanical integrity score

## Abstract

**Introduction and Hypothesis:**

The aim of this study is to develop and validate a new integral parameter, the Biomechanical Integrity score (BI-score), for the characterization of the female pelvic floor.

**Methods:**

A total of 253 subjects with normal and pelvic organ prolapse (POP) conditions were included in the multi-site observational, case-control study; 125 subjects had normal pelvic floor conditions, and 128 subjects had POP stage II or higher. A Vaginal Tactile Imager (VTI) was used to acquire and automatically calculate 52 biomechanical parameters for eight VTI test procedures (probe insertion, elevation, rotation, Valsalva maneuver, voluntary muscle contractions in two planes, relaxation, and reflex contraction). Statistical methods were applied (*t*-test, correlation) to identify the VTI parameters sensitive to the pelvic conditions.

**Results:**

Twenty-six parameters were identified as statistically sensitive to POP development. They were subdivided into five groups to characterize (1) tissue elasticity, (2) pelvic support, (3) pelvic muscle contraction, (4) involuntary muscle relaxation, and (5) pelvic muscle mobility. Every parameter was transformed to its standard deviation units against the patient age similar to T-score for bone density. Linear combinations with specified weights led to the composition of five component parameters for groups (1)–(5) and the BI-score in standard deviation units. The *p*-value for the BI-score has *p* = 4.3 × 10^−31^ for POP versus normal conditions. A reference BI-score curve against age for normal pelvic floor conditions was defined.

**Conclusions:**

Quantitative transformations of the pelvic tissues, support structures, and functions under diseased conditions may be studied with the BI-score in future research and practical applications.

## Introduction

Pelvic organ prolapse (POP) is a common condition in women and often associated with concomitant pelvic floor disorders, including urinary and fecal incontinence, pelvic pain, voiding, and sexual dysfunctions, which may adversely affect the quality of life [1]. The current clinical practice for the assessment of pelvic floor disorders is often limited to the evaluation of surface anatomy and manual palpation. The Pelvic Organ Prolapse Quantification (POP-Q) system is widely used for describing and staging pelvic support [2], the Pelvic Floor Distress Inventory (PFDI) and PFDI-20 are recommended by the International Consultation on Incontinence as grade A for assessing pelvic floor dysfunction [3], and the Female Sexual Function Index (FSFI) is used for diagnosing sexual dysfunction in women [4]. In severe or complicated cases, ultrasound, magnetic resonance imaging (MRI), and X-ray imaging may be used for additional evaluation. Bladder and rectum function tests, such as urodynamics, manometry, or defecography, might also be employed [5–8].

The true etiology of POP and variations observed among individuals are not entirely understood. These disorders are thought to share common pathogeneses, tissue elasticity changes, weakening of the connective support tissues, and pelvic muscle dysfunction [9–14]. Logically, proposing a biomechanical assessment and characterization of the female pelvic floor could give rise to important information in clinical practice. However, ultrasound and MRI elastography, as well as functional imaging of the pelvic floor, did not obtain appropriate acceptance in urogynecology. There is a significant gap in the biomechanical and functional research of the female pelvic floor [15–20]. The PUBMED database search for “(biomechanical[Title/Abstract]) AND (functional[Title/Abstract]) AND (pelvic floor[Title/Abstract])” results in a total of only 16 publications for all the time.

The Vaginal Tactile Imager (VTI) was developed to provide biomechanical mapping of the pelvic floor with a vaginal probe [21]. A set of new clinical markers/parameters has been proposed for the biomechanical characterization of the pelvic floor conditions [20, 22]. This set includes 52 parameters automatically calculated as a result of the completion of eight examination procedures (tests). However, this approach did not gain momentum among urogynecologists because of the long list of parameters and difficulties in their explanations to clinicians and patients. To make biomechanical mapping in urogynecology more accessible and useful, further work is required on the development of a shorter list of easily understandable and practical parameters.

The aim of this article is to report the development and validation of a new integral parameter, the Biomechanical Integrity score (BI-score), for the characterization of female pelvic floor conditions.

## iImaging is a medical imaging modality that translates the sense of touch into a digital imageMaterials and Methods

### Definitions

*Tactile Imaging* is a medical imaging modality that translates the sense of touch into a digital image [21]. The tactile image is a function of *P(x,y,z)*, where *P* is the pressure on the soft tissue surface under applied deformation and *x*, *y*, and *z* are the coordinates where *P* was measured. The tactile image is a pressure map on which the direction of tissue deformation must be specified.

*Functional Tactile Imaging* translates muscle activity into dynamic pressure pattern *P(x,y,t)* for an area of interest, where *t* is time and *x* and *y* are coordinates where the pressure *P* was measured. It may include: (1) muscle voluntary contraction, (2) involuntary reflex contraction, (3) involuntary relaxation, and (4) specific maneuvers.

#### Biomechanical Mapping = Tactile Imaging + Functional Tactile Imaging

A tactile imaging probe has a pressure sensor array mounted on its face that acts similarly to human fingers during a clinical examination, deforming the soft tissue and detecting the resulting changes in the pressure pattern on the surface. The sensor head is moved against or over the surface of the tissue to be studied, and the pressure response is measured at multiple locations along the tissue. The results are used to generate images that show pressure distribution over the area of the tissue under study. The tactile image *P(x,y,z)* reveals tissue or organ anatomy and elasticity distribution [23, 24].

### Vaginal Tactile Imager

**Fig. 1:**
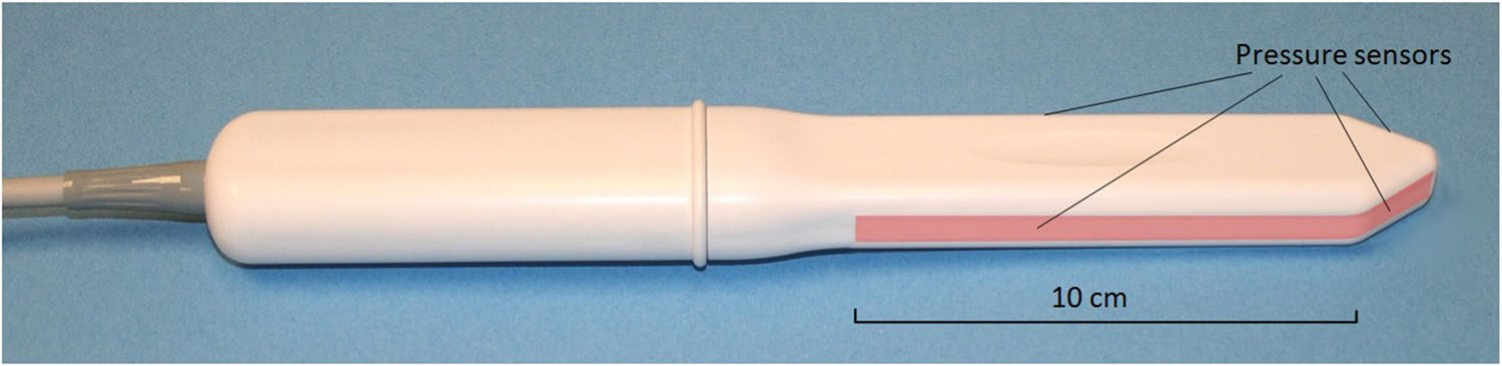
Vaginal probe. Pressure sensors are aligned on the outer surfaces of the probe (highlighted in the image)

The Vaginal Tactile Imager (VTI), model 2S, was used for biomechanical mapping of the pelvic floor. The VTI probe, as shown in Fig. 1, is equipped with 96 pressure (tactile) sensors spaced consecutively on both sides of the probe, an orientation sensor, and temperature controllers to provide the probe temperature close to a human body before the examination. During the clinical procedure, the probe is used to acquire pressure responses from two opposite vaginal walls along the vagina. The VTI data are sampled from the probe sensors and presented on the VTI display in real time. The resulting pressure maps (tactile images) of the vagina integrate all the acquired pressure and positioning data for each of the pressure sensing element during vaginal wall deformation and pelvic muscle contraction. Lubricating jel is used for patient comfort. It is also utilized to provide reproducible boundary/contact conditions with deformed tissues.

The VTI examination procedure consists of eight tests (see Appendix 1). The spatial gradients *∂P(x, y)/∂y* (changes of pressure along the tissue deformation per 1 mm) for anterior and posterior compartments are calculated within the acquired tactile images in Test 1 and 2; the *y*-coordinate is directed orthogonally from the vaginal channel coming through the anterior-posterior compartments, and the *x*-coordinate is located on the vaginal channel.

The VTI probe is calibrated with reference pressure sensors (Honeywell) immediately before every subject's examination. The VTI absolute measurement accuracy is as follows: ± 0.2 kPa within 0-10 kPa range, ± 0.5 kPa at 25 kPa, and ± 1.0 kPa at 60 kPa. The VTI relative pressure measurement accuracy lies in the range of ± 0.05 kPa to ± 0.1 kPa. The intra- and inter-observer reproducibility of the vaginal tactile imaging was reported earlier [25]. The VTI pressure measurement resolution is 0.001 kPa. The VTI absolute measurement accuracy for probe orientation is ± 0.5° and ± 0.1 °C for measuring the temperature inside the probe on the surface of the pressure sensors. The tactile images and muscle contraction patterns are visualized with a resolution of 1 mm [21].

### Biomechanical parameters

The full list of 52 VTI biomechanical parameters, their interpretation, and anatomical assignments of the targeting/contributing pelvic structures into the specified parameters are presented in Appendix 2.

### Study population

The analyzed dataset in this study includes subjects with normal pelvic floor conditions and POP stage II or higher from three VTI clinical studies with identical VTI examination procedures. These subjects were examined with the VTI in the scope multi-site observational, case-control studies completed from September 2014 to December 2018 (clinical trials identifiers NCT02294383 and NCT02925585) and study with VTI11 protocol (June 2020–September 2021). The recruitment sites were Princeton Urogynecology (Princeton, NJ), Eastern Virginia Medical School (Norfolk, VA), INOVA Fairfax Hospital (Falls Church, VA), The Institute for Female Pelvic Medicine and Reconstructive Surgery (Allentown, PA), and The Pelvic Floor Institute (Tampa, FL). It was important that all the analyzed subjects have not had any prior pelvic surgery. Table 1 presents the mean and standard deviation for the subject age, parity, weight, and height separately for normal and POP groups. All clinical protocols were approved by the Institutional Review Board (Western IRB and local IRB as required), and written informed consent was obtained from all the subjects enrolled in the studies. This clinical research was done in compliance with the Health Insurance Portability and Accountability Act. The VTI examination data for the eight tests (see Appendix 1) were obtained and recorded at the time of the scheduled urogynecologic visits.

**Table 1:** Demographic data for the studied groups

	Norm mean (*n* = 125)	Norm SD (*n* = 125)	POP mean (*n* = 128)	POP SD (*n* = 128)	100%*(POP-Norm)/Norm	Norm vs. POP *p*-value
Patient age, years	36.0	15.0	65.5	11.5	81.9%	2.6E-35
Patient parity	0.9	1.0	2.4	1.1	166.7%	2.5E-25
Patient weight, kg	69.5	14.3	70.0	12.9	0.7%	7.6E-01
Patient height, cm	163.2	9.6	162.3	7.7	−0.6%	4.4E-01
Patient POP-Q	0	–	Stage II+	–	–	–

The total study workflow comprised the following steps: (1) recruiting women who had not previously had a pelvic surgery and had normal pelvic floor conditions (no POP, stage 0) or had POP stage II or higher; (2) acquiring clinical diagnostic information related to the case by standard clinical means; (3) performing a VTI examination in lithotomic position; (4) analyzing VTI data. Study exclusion criteria were: (1) active skin infection or ulceration within the vagina; (2) presence of a vaginal septum; (3) active cancer of the colon, rectal wall, cervix, vaginal, uterus, or bladder; (4) ongoing radiation therapy for pelvic cancer; (5) impacted stool; (6) significant pre-existing pelvic pain, including levator ani syndrome, severe vaginismus, or vulvodynia; (7) severe hemorrhoids; (8) significant circulatory or cardiac conditions that could cause excessive risk from the examination as determined by the attending physician; (9) current pregnancy. In the VTI11 protocol, which targets the normal pelvic conditions, two exclusion criteria were added as follows: (1) the woman is a regular patient visiting the urogynecology clinic (2+ times during the last year); (2) the patient has cognitive impairment. Prior to the VTI examination, a standard physical examination was performed, including a bimanual pelvic examination and Pelvic Organ Prolapse Quantification (POP-Q) [2].

### Statistical methods

A total of 52 biomechanical parameters were calculated automatically by the VTI software version 2018.54.4.0 per each of the 253 analyzed VTI examination data. The two-sample *t*-test (*p* < 0.05) was employed to test the null hypothesis that the data in normal and POP groups have equal means and equal variances. The alternative hypothesis is that the data in these groups come from populations with unequal means. *P*-values for testing hypothesis were calculated. Pearson’s linear correlation coefficients (*r*) were calculated among 52 VTI parameters, each parameter against all other 51 parameters.

For the visual evaluation of the analyzed data distributions, we used notched boxplots [26] showing a confidence interval for the median value (central vertical line), 25% and 75% quartiles. The spacing between the different parts of the box helps to compare variance. The boxplot also determines skewness (asymmetry) and outlier (cross). The statistical functions of MATLAB, version R2021a (MathWorks, MA), were used for the data analysis.

## Results

Among the 253 subjects analyzed in this study, 125 had normal pelvic floor conditions and 128 had POP stage II or higher. The pelvic floor conditions were categorized by the stage of the prolapse based on the maximum stage from anterior, posterior, and uterine prolapse. Employing this approach, we found that 68 subjects had POP stage II, 57 had stage III, and 3 had stage IV.

**Fig. 2:**
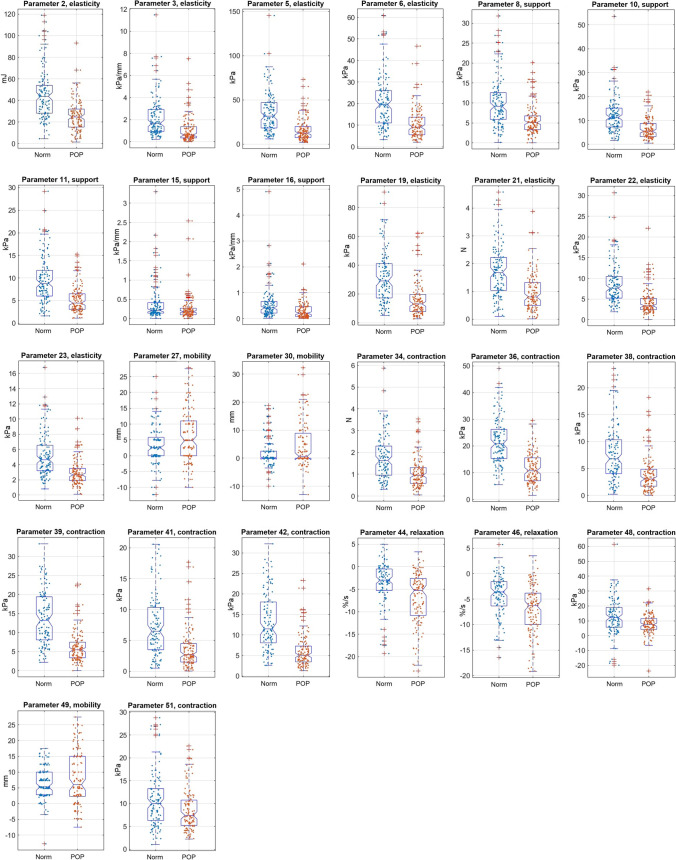
Boxplots for 26 identified VTI parameters which demonstrate statistically significant sensitivity to POP conditions and not highly correlated with each other

At first, we aimed at selecting VTI parameters with significant changes at POP versus the normal pelvic conditions. Two specific quantitative criteria were imposed on such selection: (1) a *t*-test *p* < 0.05 for the sub-set data of 128 POP cases against the sub-set data for 125 normal cases and (2) a correlation coefficient *r* < 0.85 with all other parameters. The first criteria passed 40 parameters, both first and second 26 parameters. Figure 2 presents the boxplots, and Table 2 shows the numerical data for these 26 selected VTI parameters responsive to POP and not highly correlated with each other. For consistency, the numbering of the VTI parameters in this article is kept exactly as in earlier publications [22].

**Table 2:** VTI parameters and BI-score sensitivity to POP conditions

Parameter no.*	Test no.	BI-score component	Parameter weight	Units	Norm mean (*n* = 125)	Norm SD (*n* = 125)	POP mean (*n* = 128)	POP SD (*n* = 128)	100%*(POP-Norm)/Norm	Norm vs. POP *p*-value
2	1	Elasticity	0.125	mJ	45.4	22.4	25.4	14.7	−44.1%	4.1E-15
3	1	Elasticity	0.125	kPa/mm	2.18	1.76	1.05	1.14	−51.9%	5.8E-09
5	1	Elasticity	0.125	kPa	35.7	23.1	16.0	12.6	−55.1%	3.6E-15
6	1	Elasticity	0.125	kPa	20.6	12.0	10.8	7.08	−47.5%	1.2E-13
8	2	Support	0.200	kPa	10.1	6.09	5.77	3.79	−43.3%	6.8E-11
10	2	Support	0.200	kPa	12.2	7.33	6.61	4.25	−46.0%	1.1E-12
11	2	Support	0.200	kPa	9.52	5.07	5.22	2.87	−45.2%	5.3E-15
15	2	Support	0.200	kPa/mm	0.38	0.47	0.25	0.31	−35.6%	6.8E-03
16	2	Support	0.200	kPa/mm	0.55	0.60	0.30	0.30	−45.9%	3.6E-05
19	3	Elasticity	0.125	kPa	31.4	17.7	16.1	12.3	−48.7%	5.7E-14
21	3	Elasticity	0.125	N	1.71	0.90	0.96	0.67	−43.6%	1.3E-12
22	3	Elasticity	0.125	kPa	8.66	4.90	4.26	3.01	−50.8%	8.3E-16
23	3	Elasticity	0.125	kPa	5.16	2.82	2.95	1.64	−42.9%	4.1E-13
27	4	Mobility	0.400	mm	3.26	6.17	5.83	8.54	79.0%	7.9E-03
30	4	Mobility	0.400	mm	1.71	4.97	4.56	7.86	167.0%	8.9E-04
34	5	Contraction	0.200	N	1.77	1.01	1.05	0.66	−40.3%	1.9E-10
36	5	Contraction	0.200	kPa	21.8	8.55	11.6	5.76	−46.7%	1.2E-23
38	6	Contraction	0.100	kPa	7.55	5.12	3.79	3.17	−49.8%	2.3E-11
39	6	Contraction	0.100	kPa	13.7	7.06	6.10	3.92	−55.7%	4.9E-22
41	6	Contraction	0.100	kPa	7.29	4.92	3.58	3.11	−50.9%	9.7E-12
42	6	Contraction	0.100	kPa	13.2	6.97	6.06	3.93	−54.4%	2.0E-20
44	7	Relaxation	0.500	%/s	−3.68	4.75	−6.99	5.70	90.0%	3.0E-06
46	7	Relaxation	0.500	%/s	−4.20	4.10	−6.95	4.79	65.3%	5.0E-06
48	8	Contraction	0.100	kPa	12.9	12.2	7.84	6.86	−39.4%	8.5E-05
49	8	Mobility	0.200	mm	6.55	4.95	8.01	8.55	22.4%	4.8E-02
51	8	Contraction	0.100	kPa	10.8	6.00	8.59	4.58	−20.5%	1.4E-03

*Parameter numbering as in Appendix 2

The parameters listed in Table 2 have different units (see column 5 in Table 2). The next step was to bring all the selected parameters to uniform units to allow their arithmetic combination. Among various possible options, the preference to the units of standard deviation was provided (see the explanation pertaining to such selection in the Discussion section). All VTI data were transformed according to Eq. 1 below.

1 $${Psd}_n^i=\left({Po}_n^i-{Pa}_n\right)/{SD}_n$$

Here, ${Po}_n^i$ is an original value of the *n* parameter for *i* subject; *Pa*_*n*_ is an arithmetic average of the *n* parameter for subjects aged 18–39 years in the group with normal pelvic conditions (92 out of 125 subjects); *SD*_*n *_is a standard deviation for the *n* parameter for 125 subjects in the group with normal pelvic conditions; ${Psd}_n^i$ is the transformed value of the *n* parameter for *i* subject in units of standard deviation.

**Fig. 3:**
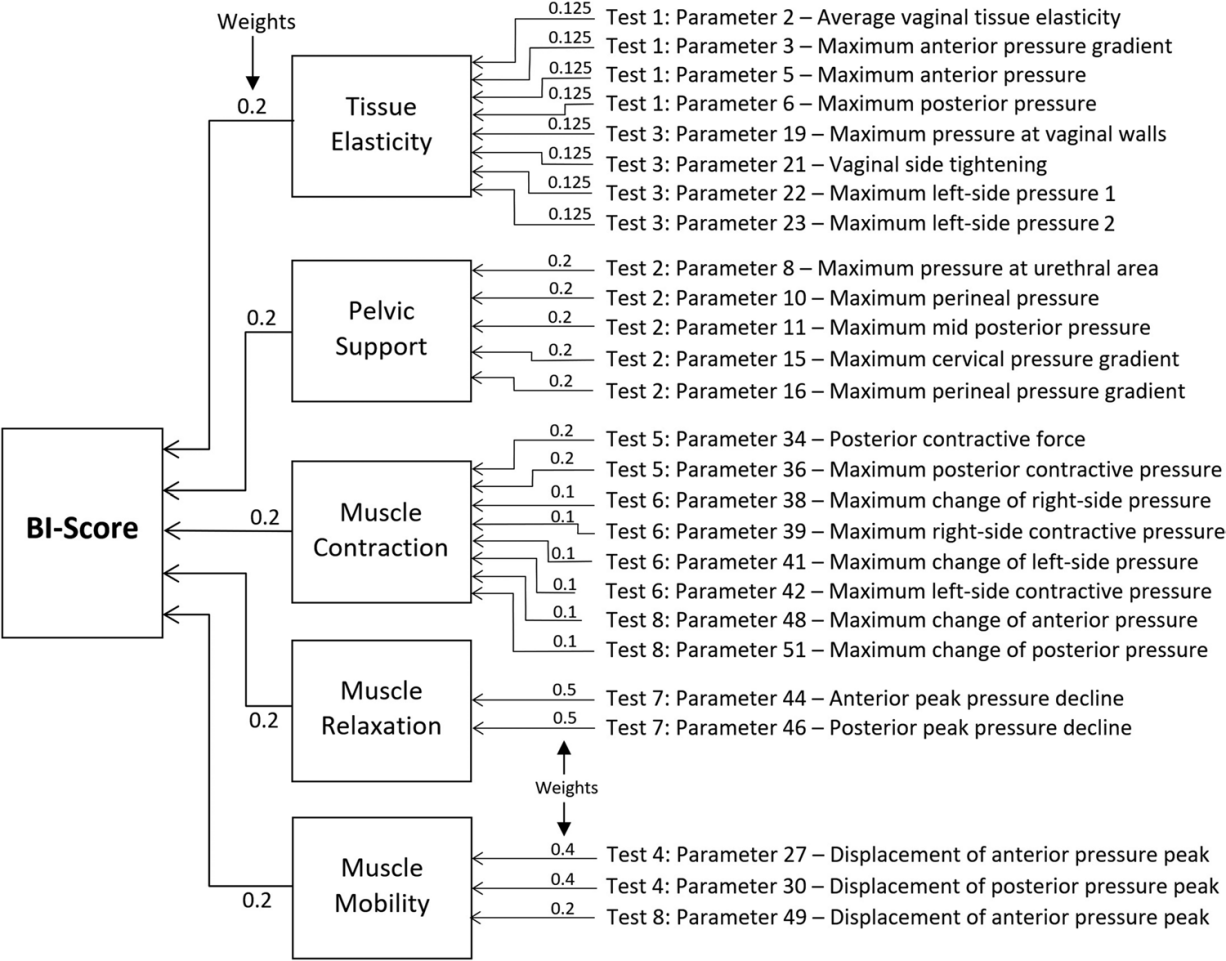
A diagram illustrating composition of BI-score from five components and VTI parameters contributing to these components with specific weights

Now, we can combine the parameters expressed in units of standard deviation using a linear operation of addition. First, the 26 selected parameters were subdivided into five groups. We may call them by the five components to characterize: (1) tissue elasticity, (2) pelvic support, (3) pelvic muscle contraction, (4) muscle relaxation, and (5) muscle mobility (see Fig. 3). Component 1 comprises eight parameters with equal weights of 0.125 (8 × 0.125 = 1.0), component 2 comprises five parameters with equal weights of 0.2, component 3 consists of eight parameters with weights of 0.1 and 0.2, component 4 comprises two parameters with weight of 0.5, and component 5 consists of three parameters with weight of 0.2 and 0.4. Basically, the same (equal) weights within the components were used to provide 1.0 at summation. In two components (muscle contraction and mobility), the preferences in weights were given to the VTI parameters with the highest *p*-values. Finally, these five components create the Biomechanical Integrity score (BI-score) with equal weights of 0.2 as shown in Fig. 3.

**Fig. 4:**
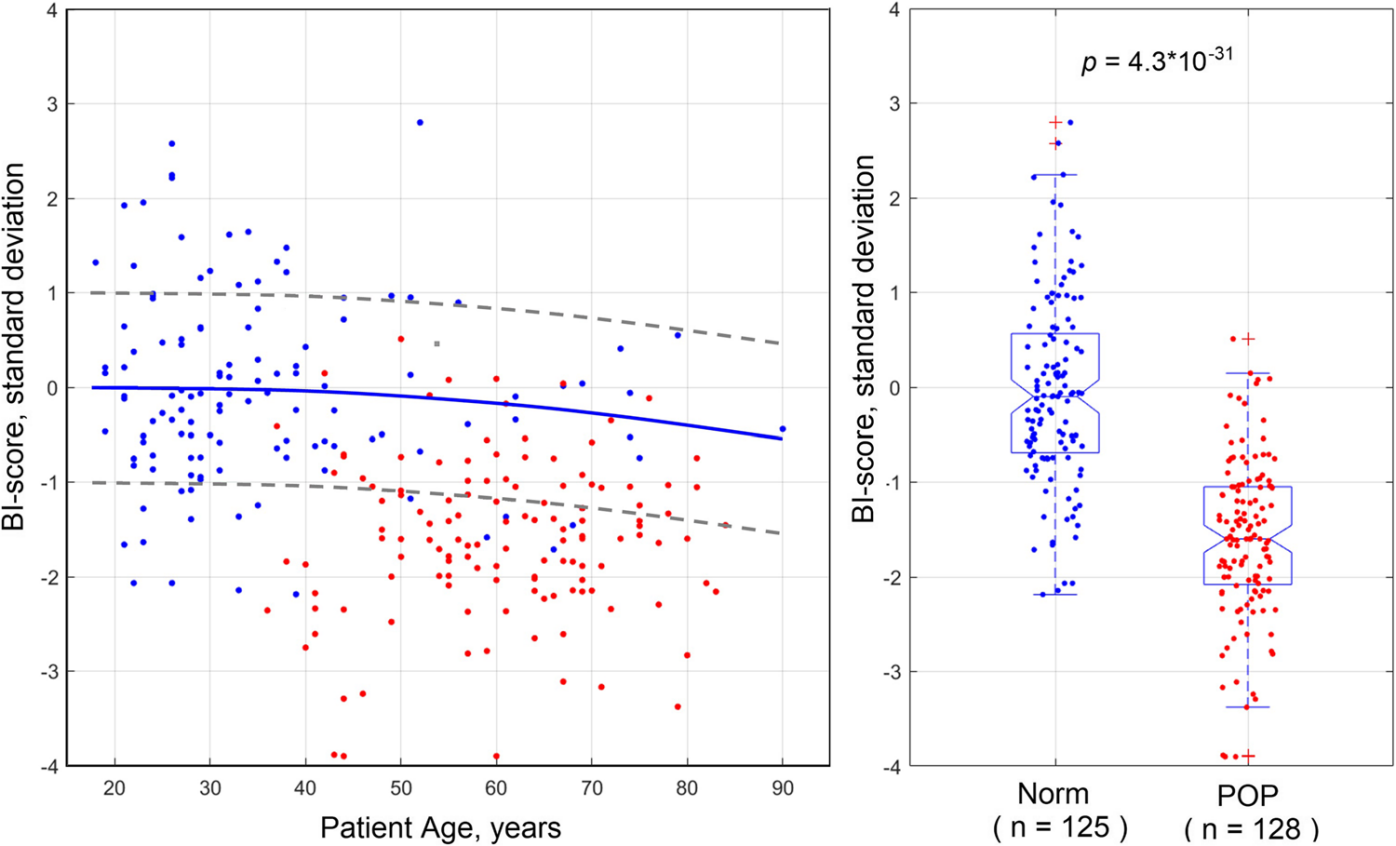
BI-score calculated for normal (blue dots) and POP (red dots) cases against patient age for 253 cases analyzed in this study (left panel). BI-score boxplots for normal (blue dots) and POP (red dots) cases

All the BI-score data for 253 subjects analyzed here can be visualized on one graph as a function of the subject’s age (see left panel in Fig. 4). A second order polynomial fit for the BI-score values against the subject’s age for all the 125 subjects in the group with normal pelvic conditions is presented by a blue line (reference line) in Fig. 4. The grey dashed lines show ± 1.0 standard deviation from the reference line. The right panel in Fig. 4 shows the same BI-score data in two boxplots for normal and POP pelvic conditions. One may observe significant separation between these two groups; the *t*-test gives *p* = 4.3 × 10^−31^ for these two groups.

**Fig. 5:**
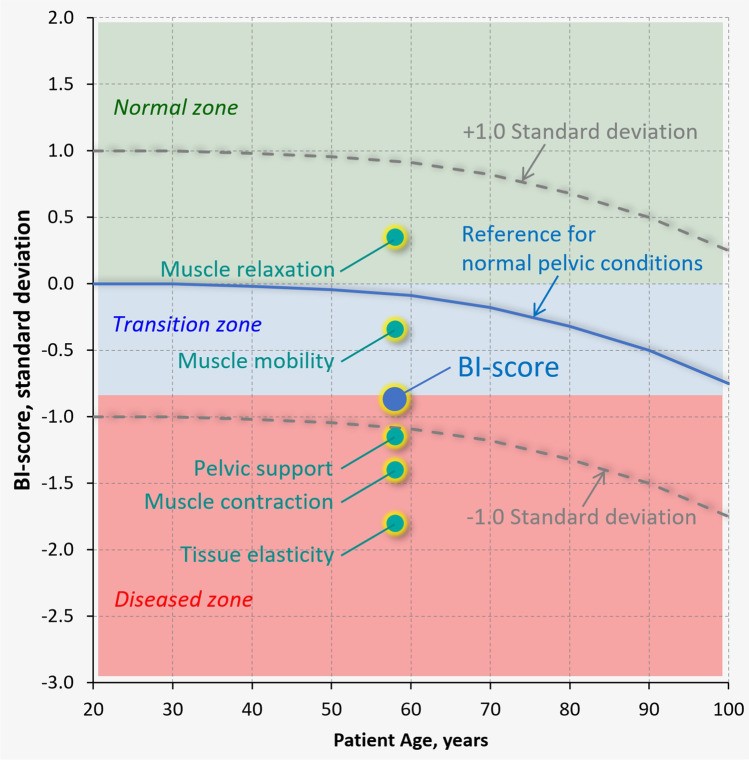
An example of examination results with BI-score and its five components for a 58-year-old patient with stage 2 anterior prolapse

Figure 5 illustrates the idea that the BI-score and all its five components can be presented on one graph with the same vertical axis (standard deviation) because all of them have the same units. Three colored backgrounds denote the zones of certainly normal pelvic conditions (green), transition (blue), and POP (red).

## Discussion

The BI-score has certain similarities with the T-score developed for bone density characterization. As bone mineral density (BMD) technology evolved in the twentieth century, it became clear that BMD expressed in raw units would be difficult to interpret. Ideally, for BMD measurements to be clinically useful, they should be presented in terms that are readily understandable by patients and clinicians as well as independent of the densitometer used or the skeletal site measured. The T-score was suggested to simplify the interpretation of the bone density result and to avoid the use of raw BMD values. The T-score was defined as the difference between a BMD and the expected young normal value divided by the population standard deviation; it is similar to Eq. 1 in this article. In a rare moment of scientific convergence among instrument manufacturers, the T-score was quickly adopted as a consistent output parameter for all densitometry devices [27]. With the World Health Organization (WHO) Study Group report, the role of the T-score for osteoporosis diagnosis was solidified [28]. Currently, osteoporosis researchers and clinicians use the T-score as the most relevant diagnostic value from a bone density examination.

The BI-score is a composite score that consists of five components as shown in Fig. 3. These five components bring different aspects of biomechanical characterization of the pelvic floor. Due to the exclusion of the highly correlated original VTI parameters with *r* ≥ 0.85, the mutual correlation coefficients have an average value of *r* = 0.27, which is considered as low or negligible correlation. It is important to note that the tissue elasticity component integrates the tissue/structure elasticity for the 0–8 mm layer behind the vaginal walls, from the depth comparative with the vaginal wall deformations in Test 1 and 3 (see Fig. 3 and parameter interpretation in Appendix 2 and [22]). The pelvic support component integrates the structure support from a depth of 5–45 mm, which is about the same as the vaginal wall deformations in Test 2 (see Fig. 3 and parameter interpretation in Appendix 2 and [27]).

Earlier, the intra- and inter-observer reproducibility for a set of ten vaginal tactile imaging markers was reported on 12 subjects [25]. Intra-observer intraclass correlation coefficients (ICC) were found in the range from 0.80 (Test 8: cough) to 0.92 (Test 3: rotation) with average value of 0.87. Inter-observer ICCs were found in the range from 0.73 (Test 2, elevation pressure, and Test 8: cough) to 0.92 (Test 3: rotation) with average value of 0.82. Intra-observer limits of agreement were in the range from ± 11.3% (Test 1) to ± 19.0%% (Test 8) with average value of ± 15.1%. Inter-observer limits of agreement were in the range from ± 12.0% (Test 5: voluntary contraction) to ± 26.7% (Test 2: elevation) with average value of ± 18.4%. These numbers lead to projection of reproductivity for the BI-score and its components in the range from ± 0.1 to ± 0.2 standard deviation. Improved inter-observer reproducibility is possible by additional operator training and consistency in VTI examination technique.

The tenth column in Table 2 shows VTI parameter changes in POP relative to the normal pelvic conditions. The elasticity parameters are decreasing by −42.9%…–55.1% in POP, the pelvic support parameters are decreasing by −35.6%…–46.0%, and the muscle contraction parameters are decreasing by −40.3%…–55.7% in POP. The muscle relaxation speed parameters, which have a negative sign because muscle force involuntarily goes down, are increasing by 65.3% and 90.0% in POP—relaxation develops faster. The muscle mobility parameters, which may have a negative or positive sign, are increasing by 79.0% and 167.0% in POP—muscle mobility develops along the vagina. All these results are expected and are in line with the previously reported results [18, 21, 22].

The mean subject age and parity in the normal and POP groups are significantly different: 36 versus 65.5 years old and 0.9 versus 2.4, respectively. That is the envisioned difference in the analyzed groups because for the reference (zero-line in the BI-score) we need a young population without POP which develops with age. Age-matched analysis for all normal versus all POP subjects aged from 38 to 52 years (*p* = 0.48 for age distributions) demonstrated *t*-test outcome for BI-score sensitivity to POP conditions with *p* = 2.1 × 10^−7^. The mean subject weight and height are the same in both groups (see Table 1).

The last column in Table 2 brings *p*-values for the two-sample *t*-tests (normal versus POP). The *p*-values for the VTI parameters are found in the range of 1.2 × 10^−23^ to 4.8 × 10^−2^, most of the *p*-values being < 1.0 × 10^−5^. The *p*-value for the BI-score has *p* = 4.3 × 10^−31^ for two analyzed groups. It indicates that the data in these groups come from populations with unequal means and strong sensitivity to POP conditions. These results can be considered as statistically significant validation for the BI-score sensitivity to POP conditions. Since POP is often associated with concomitant pelvic floor disorders, including urinary and fecal incontinence, pelvic pain, voiding, and sexual dysfunctions [1, 29], and these disorders are thought to share common pathogeneses, tissue elasticity changes, weakening of the connective support tissues, and pelvic floor muscle dysfunction [9–14], the proposed BI-score may be used for the characterization of any of the above listed pelvic disorders and/or their combination.

Three colored backgrounds have been suggested to be used in the presentation of the patient examination results as shown in Fig. 5. The transition from green to blue background at BI-score = 0 has sensitivity = 95.3% and specificity = 51.2% for the detection of POP conditions. The transition from blue to red background at BI-score = −0.80 has almost equalized sensitivity = 82.8% and specificity = 84.0% for diagnosing POP conditions. These transitions (BI-score = 0 and BI-score = −0.80) are present in Fig. 5. The POP diagnostic accuracy of the BI-score, calculated as an area under a receiver operating characteristics (ROC) curve [30] for the analyzed sample, was found as 89.7%. The dependence of the BI-score on age for normal pelvic conditions is described as a second order curve (see blue line in Fig. 5); its ± standard deviations are depicted by the dashed curves. It is clear that an age-adjusted BI-score can also be calculated relatively to the normal blue curve in Fig. 5 similar to the Z-score in the bone densitometry.

As with bone density measurement, it is important to monitor patient progress with or without treatment. For this reason, it would be important to define the minimal clinically important difference (MCID) in BI-score. The future research directions also may address (1) BI-score use for monitoring of a pelvic floor treatment outcome, (2) obtaining periodic BI-scores before a woman has symptoms, (3) recommendation for specific treatment based on the five components (e.g., treatment for elasticity is needed but not for relaxation or muscle mobility), (4) predictive capabilities of the BI-score for symptoms (e.g., a woman is less or more likely to develop some form of pelvic floor dysfunction). These important questions are beyond the scope of this article.

The strength of this study is that the suggested BI-score covers biomechanical aspects of the pelvic floor, which include tissue elasticity, pelvic support, muscle contraction, involuntary relaxation, and mobility. All these aspects usually deteriorate as the pelvic disease develops. This quantitative characterization can be used in diagnosing and monitoring the pelvic conditions as well as in selecting and justifying a treatment.

The weakness of this study is the absence of statistically significant results for possible variations with ethnicity and race, which must be the subject of future research. Also, thousands of new VTI examinations for normal pelvic floor conditions may adjust the values of mean and standard deviation used in Eq. 1 for BI-score calculations.

## Conclusion

The original purpose of this research was to systemize and simplify the presentation of the VTI examination results. Based on the analysis, it can be concluded that there are five components important for the biomechanical characterization of the pelvic floor. All these components contribute to the integral parameter the BI-score. Objectively measurable transformations of the pelvic tissues, support structures, and functions under different diseased conditions may be studied with the BI-score in future research and practical applications.

## Appendix 1.

Table 3

**Table 3:** VTI examination procedure tests

Test no.	Procedure	Output
Test 1	Probe insertion	Tactile image* for vaginal anterior and posterior compartments along the entire vagina (resistance, force, work, pressure gradients, tissue elasticity); 3–15-mm tissue deformation
Test 2	Probe elevation	Tactile image* for anterior and posterior compartments, which related to pelvic floor support structures (pressure value and pressure gradients for specified/critical locations); 20–45-mm tissue deformation
Test 3	Probe rotation	Tactile images* for left and right sides along the entire vagina (force and pressure values for specified locations); 5–7-mm tissue deformation
Test 4	Valsalva maneuver	Dynamic pressure response from opposite compartments (anterior vs. posterior) along the entire vagina (changes in force and pressure; pressure peak displacements).
Test 5	Voluntary muscle contraction	Dynamic pressure response from opposite compartments (anterior vs. posterior) along the entire vagina (changes in force and pressure; maximum pressure values)
Test 6	Voluntary muscle contraction	Dynamic pressure response from opposite sides (left vs. right) along the entire vagina (changes in force and pressure; maximum pressure values)
Test 7	Involuntary relaxation	Dynamic pressure response from opposite compartments (anterior vs. posterior) along the entire vagina (changes in pressure)
Test 8	Reflex muscle contraction (cough)	Dynamic pressure response from opposite compartments (anterior vs. posterior) along the entire vagina (changes in force and pressure; pressure peak displacements).

*Probe maneuvers in Tests 1–3 are used to accumulate multiple pressure patterns from the tissue surface and create an integrated tactile image for the investigated area employing the image composition algorithms described in [23, 24]

## Appendix 2

Table 4

**Table 4:** VTI biomechanical parameters

No.	VTI test	Parameters abbreviation	Units	Parameter description	Parameter interpretation	Parameter class	Targeting/contributing pelvic structures
1	1	Fmax	N	Maximum value of force measured during the VTI probe insertion	Maximum resistance of anterior vs. posterior widening; tissue elasticity at specified location (capability to resist to applied deformation)	Maximum vaginal tissue elasticity at specified location	Tissues behind the anterior and posterior vaginal walls at 3–15-mm depth
2	1	Work	mJ	Work completed during the probe insertion (work = force × displacement)	Integral resistance of vaginal tissue (anterior and posterior) along the probe insertion	Average vaginal tissue elasticity	Tissues behind the anterior and posterior vaginal walls at 3–15-mm depth
3	1	Gmax_a	kPa/mm	Maximum value of anterior gradient (change of pressure per anterior wall displacement in orthogonal direction to the vaginal channel)	Maximum value of tissue elasticity in anterior compartment behind the vaginal at specified location	Maximum value of anterior tissue elasticity	Tissues/structures in anterior compartment at 10–15-mm depth
4	1	Gmax_p	kPa/mm	Maximum value of posterior gradient (change of pressure per posterior wall displacement in orthogonal direction to the vaginal channel)	Maximum value of tissue elasticity in posterior compartment behind the vaginal at specified location	Maximum value of posterior tissue elasticity	Tissues/structures in anterior compartment at 10–15-mm depth
5	1	Pmax_a	kPa	Maximum value of pressure per anterior wall along the vagina	Maximum resistance of anterior tissue to vaginal wall deformation	Anterior tissue elasticity	Tissues/structures in anterior compartment
6	1	Pmax_p	kPa	Maximum value of pressure per posterior wall along the vagina	Maximum resistance of posterior tissue to vaginal wall deformation	Posterior tissue elasticity	Tissues/structures in posterior compartment
7	2	P1max_a	kPa	Maximum pressure at the area of pubic bone	Proximity of pubic bone to vaginal wall and perineal body strength	Anatomic aspects and tissue elasticity	Tissues between vagina and pubic bone; perineal body
8	2	P2max_a	kPa	Maximum pressure at the area of urethra	Elasticity/mobility of urethra	Anatomic aspects and tissue elasticity	Urethra and surrounding tissues
9	2	P3max_a	kPa	Maximum pressure at the cervix area	Mobility of uterus and conditions of uterosacral and cardinal ligaments	Pelvic floor support	Uterosacral and cardinal ligaments
10	2	P1max_p	kPa	Maximum pressure at the perineal body	Pressure feedback of level III support	Pelvic floor support	Puboperineal, puborectal muscles
11	2	P2max_p	kPa	Maximum pressure at middle third of vagina	Pressure feedback of level II support	Pelvic floor support	Pubovaginal, puboanal muscles
12	2	P3max_p	kPa	Maximum pressure at upper third of vagina	Pressure feedback of level I support	Pelvic floor support	Iliococcygeal muscle, levator plate
13	2	G1max_a	kPa/mm	Maximum gradient at the area of pubic bone	Vaginal elasticity at pubic bone area	Anterior tissue elasticity	Tissues between vagina and pubic bone; perineal body
14	2	G2max_a	kPa/mm	Maximum gradient at the area of urethra	Mobility and elasticity of urethra	Urethral tissue elasticity	Urethra and surrounding tissues
15	2	G3max_a	kPa/mm	Maximum gradient at the cervix area	Conditions of uterosacral and cardinal ligaments	Pelvic floor support	Uterosacral and cardinal ligaments
16	2	G1max_p	kPa/mm	Maximum gradient at the perineal body	Strength of level III support (tissue deformation up to 25 mm)	Pelvic floor support	Puboperineal, puborectal muscles
17	2	G2max_p	kPa/mm	Maximum gradient at middle third of vagina	Strength of level II support (tissue deformation up to 35 mm)	Pelvic floor support	Pubovaginal, puboanal muscles
18	2	G3max_p	kPa/mm	Maximum gradient at upper third of vagina	Strength of level I support (tissue deformation up to 45 mm)	Pelvic floor support	Iliococcygeal muscle, levator plate
19	3	Pmax	kPa	Maximum pressure at vaginal wall deformation by 7 mm	Hard tissue or tight vagina	Vaginal tissue elasticity	Tissues behind the vaginal walls at 5–7-mm depth
20	3	Fap	N	Force applied by anterior and posterior compartments to the probe	Integral strength of anterior and posterior compartments	Vaginal tightening	Tissues behind anterior/ posterior vaginal walls
21	3	Fs	N	Force applied by entire left and right sides of vagina to the probe	Integral strength of left and right sides of vagina	Vaginal tightening	Vaginal right/left walls and tissues behind them
22	3	P1_l	kPa	Pressure response from a selected location (irregularity 1) at left side	Hard tissue on left vaginal wall	Irregularity on vaginal wall	Tissue/muscle behind the vaginal walls on left side.
23	3	P2_l	kPa	Pressure response from a selected location (irregularity 2) at left side	Hard tissue on left vaginal wall	Irregularity on vaginal wall	Tissue/muscle behind the vaginal walls on left side
24	3	P3_r	kPa	Pressure response from a selected location (irregularity 3) at right side	Hard tissue on right vaginal wall	Irregularity on vaginal wall	Tissue/muscle behind the vaginal walls on right side
25	4	dF_a	N	Integral force change in anterior compartment at Valsalva maneuver	Pelvic function* at Valsalva maneuver	Pelvic function	Multiple pelvic muscle*
26	4	dPmax_a	kPa	Maximum pressure change in anterior compartment at Valsalva maneuver	Pelvic function* at Valsalva maneuver	Pelvic function	Multiple pelvic muscle*
27	4	dL_a	mm	Displacement of the maximum pressure peak in anterior compartment	Mobility of anterior structures*Valsalva maneuver	Pelvic function	Urethra, pubovaginal muscle; ligaments*
28	4	dF_p	N	Integral force change in posterior compartment at Valsalva maneuver	Pelvic function* at Valsalva maneuver	Pelvic function	Multiple pelvic muscle*
29	4	dPmax_p	kPa	Maximum pressure change in posterior compartment at Valsalva maneuver	Pelvic function* at Valsalva maneuver	Pelvic function	Multiple pelvic muscle*
30	4	dL_p	mm	Displacement of the maximum pressure peak in posterior compartment	Mobility of posterior structures*Valsalva maneuver	Pelvic function	Anorectal, puborectal, pubovaginal muscles; ligaments*
31	5	dF_a	N	Integral force change in anterior compartment at voluntary muscle contraction	Integral contraction strength of pelvic muscles along the vagina	Pelvic function	Puboperineal, puborectal, pubovaginal, and ilicoccygeal muscles; uretra
32	5	dPmax_a	kPa	Maximum pressure change in anterior compartment at voluntary muscle contraction	Contraction strength of specified pelvic muscles	Pelvic function	Puboperineal, puborectal and pubovaginal muscles
33	5	Pmax_a	kPa	Maximum pressure value in anterior compartment at voluntary muscle contraction	Static and dynamic peak support of the pelvic floor	Pelvic function	Puboperineal and puborectal muscles*
34	5	dF_p	N	Integral force change in posterior compartment at voluntary muscle contraction	Integral contraction strength of pelvic muscles along the vagina	Pelvic function	Puboperineal, puborectal, pubovaginal, and ilicoccygeal muscles
35	5	dPmax_p	kPa	Maximum pressure change in posterior compartment at voluntary muscle contraction	Contraction strength of pelvic muscles at specified location	Pelvic function	Puboperineal, puborectal and pubovaginal muscles
36	5	Pmax_p	kPa	Maximum pressure value in posterior compartment at voluntary muscle contraction	Static and dynamic peak support of the pelvic floor	Pelvic function	Puboperineal and puborectal muscles*
37	6	dF_r	N	Integral force change in right side at voluntary muscle contraction	Integral contraction strength of pelvic muscles along the vagina	Pelvic function	Puboperineal, puborectal, and pubovaginal muscles
38	6	dPmax_r	kPa	Maximum pressure change in right side at voluntary muscle contraction	Contraction strength of specific pelvic muscle	Pelvic function	Puboperineal or puborectal or pubovaginal muscles
39	6	Pmax_r	kPa	Maximum pressure value in right side at voluntary muscle contraction	Specified pelvic muscle contractive capability and integrity	Pelvic function	Puboperineal or puborectal muscles
40	6	dF_l	N	Integral force change in left side at voluntary muscle contraction	Integral contraction strength of pelvic muscles along the vagina	Pelvic function	Puboperineal, puborectal, and pubovaginal muscles
41	6	dPmax_l	kPa	Maximum pressure change in left side at voluntary muscle contraction	Contraction strength of specific pelvic muscle	Pelvic function	Puboperineal or puborectal or pubovaginal muscles
42	6	Pmax_l	kPa	Maximum pressure value in left side at voluntary muscle contraction	Specified pelvic muscle contractive capability and integrity	Pelvic function	Puboperineal or puborectal muscles
43	7	dPdt_a	kPa/s	Anterior absolute pressure change per second for maximum pressure at involuntary relaxation	Innervation status of specified pelvic muscles	Innervations status	Levator ani muscles
44	7	dpcdt_a	%/s	Anterior relative pressure change per second for maximum pressure at involuntary relaxation	Innervation status of specified pelvic muscles	Innervations status	Levator ani muscles
45	7	dPdt_p	kPa/s	Posterior absolute pressure change per second for maximum pressure at involuntary relaxation	Innervation status of specified pelvic muscles	Innervations status	Levator ani muscles
46	7	dpcdt_p	%/s	Posterior relative pressure change per second for maximum pressure at involuntary relaxation	Innervation status of specified pelvic muscles	Innervations status	Levator ani muscles
47	8	dF_a	N	Integral force change in anterior compartment at reflex pelvic muscle contraction (cough)	Integral pelvic function* at reflex muscle contraction	Pelvic function	Multiple pelvic muscle*
48	8	dPmax_a	kPa	Maximum pressure change in anterior compartment at reflex pelvic muscle contraction (cough)	Contraction strength of specified pelvic muscles	Pelvic function	Multiple pelvic muscle*
49	8	dL_a	mm	Displacement of the maximum pressure peak in anterior compartment	Mobility of anterior structures* at reflex muscle contraction	Pelvic function	Urethra, pubovaginal muscle; ligaments*
50	8	dF_p	N	Integral force change in posterior compartment at reflex pelvic muscle contraction (cough)	Integral pelvic function* at reflex muscle contraction	Pelvic function	Multiple pelvic muscle*
51	8	dPmax_p	kPa	Maximum pressure change in posterior compartment at reflex pelvic muscle contraction (cough)	Contraction strength of specified pelvic muscles	Pelvic function	Multiple pelvic muscle*
52	8	dL_p	mm	Displacement of the maximum pressure peak in posterior compartment	Mobility of anterior structures* at reflex muscle contraction	Pelvic function	Anorectal, puborectal and pubovaginal muscles; ligaments*

*Requires further interpretation
